# Is it possible to predict optimal rocker shoe design using barefoot gait parameters?

**DOI:** 10.1186/1757-1146-7-S1-A65

**Published:** 2014-04-08

**Authors:** Jonathan D Chapman, Stephen J Preece, Christopher J Nester, Bjoern Braunstein, Angela Höhne, Gert-Peter Brüggermann

**Affiliations:** 1 School of Health, Sport and Rehabilitation Sciences, University of Salford, UK; 2 Institute of Biomechanics and Orthopaedics, German Sport University, Cologne, Germany

## Background

Curved rocker shoes are routinely prescribed for people with diabetes in order reduce in-shoe plantar pressures. However, previous research has shown that different individuals may require different rocker outsole geometries in order to optimise pressure reduction [1,2]. This has led some researchers to suggest that every individual should try a range of possible outsole designs to identify the design which maximises pressure reduction [1]. However, this process may not be feasible in a clinical setting. Given that plantar pressure has been shown to depend on specific gait variables [3], it may be possible to develop an algorithm which could predict an individual’s pressure response to a specific rocker outsole design using an input of gait data. Such an algorithm would remove the need to try on a large number of pairs of rocker shoes.

## Objective

To investigate the accuracy of an algorithm developed to predict peak plantar pressure for eight different rocker shoes designs from an input of barefoot gait data.

## Methods

The eight rocker shoes designs spanned different combinations of two design features: rocker angle (15° or 20°) and apex position (52%, 57%, 62%, 67% shoe length). A total of n=76 patients were recruited into the study and each participant wore each of the eight shoes whilst foot pressure was measured during walking. A gait assessment was then carried out as the participant walked barefoot and a set of gait and anthropometric variables defined as algorithm inputs. A separate algorithm was then developed to predict peak plantar pressure for each of the eight shoes in three different forefoot regions. In order to develop each algorithm, a regression approach was first used identify a suitable subset of inputs and to estimate the percentage of the variance in peak pressure explained by the inputs. A neural network was then developed and tested to assess predictive power.

## Results

The regression analysis showed that it was possible to explain between 21% and 47% of the variance in peak pressure, typically with a set of 3-6 gait/anthropometric variables. However, the predictive power of the neural networks was relatively low, between 24-49%

## Conclusion

Although the results demonstrated clear correlations between groups of gait/anthropometric variables and peak pressure, the predictive power of the algorithm was not high enough for routine use in clinical practice. This may be because additional input variables, such as bony geometry are required to improve algorithm accuracy.
